# Supplementary material for: Hepatocellular Carcinoma: Old and Emerging Therapeutic Targets
Source: Cancers (Basel). 2024 Feb 23;16(5):901. doi: 10.3390/cancers16050901 (PMC10931414; doi:10.3390/cancers16050901)
Supplement: Supplementary file 1 [file cancers-16-00901-s001.zip › cancers-2877364-supplementary.pdf]

ImmunoHUB Author List:

M. Terreni[1], T. Bavaro[1], M. Serra[1], P. Linciano[1], D. Rubes[1], C. Lanni[1], C. Travelli[1], M. Paolillo[1], A. Nicole[1], C. Temporini[1], S. Tengattini[1], F. Rinaldi[1], G. Massolini[1], E. De Lorenzi[1], S. Rossi[1], G. Sandri[1], B. Vigani[1], M. C. Bonferoni[1], B. Conti[1], I. Genta[1], R. Dorati[1], E. Chiesa[1], S. Perteghella[1], C. Scotti[2], M. Maggi[2], G. Pessino[2], S. Calandra[2], H. de Jonge[2], L. Iamele[2], C. Turato[2], S. de Siervi[2], M. Lolicato[2], G. Colombo[3], F. Doria[3], S. Serapian[3], V. Pirota[3], F. Corana[4], B. Mannucci[4], F. Forneris[5,6], Francesca Magnani[5], S. Liberi[5], A. Canciani[5], S. Faravelli[5], F. Lescai[5], M. G. Bottone[5], M. Peviani[5], P. Rossi[5], M.Torti[5], P. Cabras[5], C. Casali[5], C. Priori[5], L. Gaiaschi[5], F. De Luca[5], E. Pelloni[5], G. Guidetti[5], F. Giammella[5], M. Mattei[7], M. Piacentini[7], R. Nisticò[7], S. Gonfloni[7], D. Mango[7], A. D'Ettorre[7], G. Melino[8], E. Candi[8], A. Mauriello[8], S. Manuel[8], R. Cicconi[9], S. V. Boccadamo Pompili[9], R. Bernardini[10], M. A. Bianca[10], L. Pollegioni[11], L. Caldinelli[11], F. Berini[11], E. Rosini[11], G. Molla[11], L. Piubelli[11], S. Sacchi[11], M. Crespi[11], E. Mascheroni[11], E. Binda[11], F. Marinelli[11], P. Battaglia[11], G. Forlani[12], M. Shallak[12], L. Azzi[12], D. Dalla Gasperina[12], F. Dentali[12], N. Mancini[12], P. A. Grossi[13], M. Fasano[14], H. Bondi[14], M. U. Mondelli[15,16], B. Oliviero[15], S. Mantovani[15], S. Varchetta[15], G. Donetti[16], F. Baldanti[17,18], I. Cassaniti[17,18], A. Piralla[17], S. Paolucci[17], F. Zavaglio[17], J. C. Sammartino[18], C. Perrotta[19], D. Trabattoni[19], E. Clementi[19,20], S. Antinori[19,21], D. Cervia[22], F. Vergani[23], E. Verzoni[23], M. Stellato[23], G. Procopio[23], A. Cattaneo[24], F. Raimondi[24], S. Lisi[24], A. Viegi[24], A. Jacob[24], M. Di Domenico[24].

1. Department of Drug Science, University of Pavia, 27100, Pavia, Italy.
2. Department of Molecular Medicine, University of Pavia, 27100, Pavia, Italy.
3. Department of Chemistry, University of Pavia, 27100, Pavia, Italy.
4. Centro Grandi Strumenti, University of Pavia, Via Bassi 21, 27100, Pavia, Italy.
5. Department of Biology and Biotechnology, University of Pavia, 27100, Pavia, Italy.
6. Fondazione IRCCS Policlinico San Matteo, 27100, Pavia, Italy,
7. Department Biology, University of Rome Tor Vergata, via della Ricerca Scientifica, Rome, Italy.
8. Department of Experimental Medicine, University of Rome Tor Vergata, Rome, Italy.
9. Centro Interdipartimentale-CIMETA, University of Tor Vergata, Rome, Italy.
10. Department of Translational Medicine, University of Tor Vergata, Rome, Italy
11. Department of Biotechnolgy and Life Science, University of Insubria, Insubria, Varese, 21100, Italy
12. Department of Medicine and Technological Innovation, University of Insubria, Insubria, Varese, 21100, Italy
13. Department of Medicine and Surgery, University of Insubria, Insubria, Varese, 21100, Italy
14. Department of High Technology Sciences, University of Insubria, Insubria, Varese, 21100, Italy
15. Division of Clinical Immunology and Infectious Diseases, Fondazione IRCCS Policlinico San Matteo, 27100 Pavia, Italy
16. Department of Internal Medicine and Therapeutics, University of Pavia, 27100, Pavia, Italy
17. Microbiology and Virology Department, Fondazione IRCCS Policlinico San Matteo, 27100, Pavia, Italy
18. Department of Clinical Surgical, Diagnostics and Pediatric Sciences, University of Pavia, 27100, Pavia, Italy
19. Department of Biomedical and Clinical Sciences (DIBIC), Università of Milan, Milan, Italy
20. Pharmacovigilance & Clinical Research, International Centre for Pesticides and Health Risk Prevention, ASST Fatebenefratelli Sacco Hospital, Milan, Italy
21. III Division of Infectious Diseases, ASST Fatebenefratelli Sacco Hospital, Milan, Italy
22. Department for Innovation in Biological, Agro-Food and Forest Systems (DIBAF), Università degli Studi della Tuscia, Viterbo, Italy
23. Medical Oncology Unit, Fondazione IRCCS Istituto Nazionale dei Tumori di Milano, Milan, Italy
24. Bio@SNS Laboratory, Scuola Normale Superiore P.zza dei Cavalieri 7, 56126 Pisa, Italy
